# Identification and characterization of two drug-like fragments that bind to the same cryptic binding pocket of *Burkholderia pseudomallei* DsbA

**DOI:** 10.1107/S2059798321011475

**Published:** 2022-01-01

**Authors:** Guillaume A. Petit, Biswaranjan Mohanty, Róisín M. McMahon, Stefan Nebl, David H. Hilko, Karyn L. Wilde, Martin J. Scanlon, Jennifer L. Martin, Maria A. Halili

**Affiliations:** a Griffith Institute for Drug Discovery, Griffith University, Building N75, 46 Don Young Road, Nathan, QLD 4111, Australia; b Medicinal Chemistry, Monash Institute of Pharmaceutical Sciences, Monash University, 381 Royal Parade, Parkville, VIC 3052, Australia; c ARC Centre for Fragment-Based Design, Monash Institute of Pharmaceutical Sciences, Monash University, 381 Royal Parade, Parkville, VIC 3052, Australia; d Sydney Analytical Core Research Facility, The University of Sydney, Sydney, NSW 2006, Australia; e National Deuteration Facility, Australian Nuclear Science and Technology Organization (ANSTO), New Illawarra Road, Lucas Heights, NSW 2234, Australia; f Vice-Chancellor’s Unit, University of Wollongong, Building 36, Wollongong, NSW 2522, Australia

**Keywords:** DsbA, fragments, cryptic pocket, *Burkholderia pseudomallei*, NMR, X-ray crystallography

## Abstract

Disulfide-bond-forming proteins (Dsbs) play a crucial role in the pathogenicity of many Gram-negative bacteria. Disulfide-bond-forming protein A (DsbA) catalyzes the formation of the disulfide bonds necessary for the activity and stability of multiple substrate proteins, including many virulence factors. Hence, DsbA is an attractive target for the development of new drugs to combat bacterial infections. Here, two fragments, bromophenoxy propanamide (**1**) and 4-methoxy-*N*-phenylbenzenesulfonamide (**2**), were identified that bind to DsbA from the pathogenic bacterium *Burkholderia pseudomallei*, the causative agent of melioidosis. The crystal structures of oxidized *B. pseudomallei* DsbA (termed BpsDsbA) co-crystallized with **1** or **2** show that both fragments bind to a hydrophobic pocket that is formed by a change in the side-chain orientation of Tyr110. This conformational change opens a ‘cryptic’ pocket that is not evident in the apoprotein structure. This binding location was supported by 2D-NMR studies, which identified a chemical shift perturbation of the Tyr110 backbone amide resonance of more than 0.05 p.p.m. upon the addition of 2 m*M* fragment **1** and of more than 0.04 p.p.m. upon the addition of 1 m*M* fragment **2**. Although binding was detected by both X-ray crystallography and NMR, the binding affinity (*K*_d_) for both fragments was low (above 2 m*M*), suggesting weak interactions with BpsDsbA. This conclusion is also supported by the crystal structure models, which ascribe partial occupancy to the ligands in the cryptic binding pocket. Small fragments such as **1** and **2** are not expected to have a high energetic binding affinity due to their relatively small surface area and the few functional groups that are available for intermolecular interactions. However, their simplicity makes them ideal for functionalization and optimization. The identification of the binding sites of **1** and **2** to BpsDsbA could provide a starting point for the development of more potent novel antimicrobial compounds that target DsbA and bacterial virulence.

## Abstract 2

The binding properties of two drug-like fragments to a conformationally dynamic site in disulfide bond-forming protein A from *B. pseudomallei* are described.

## 1. Introduction

Fragment-based drug discovery (FBDD) is a method used to develop potent small-molecule compounds against a target protein or enzyme starting from simple building-block molecules called fragments. Fragments often bind with low affinity due to their small size and therefore form few interactions with the protein. However, the combination and/or modification of these simple building blocks can lead to potent compounds (Murray & Rees, 2009 ▸; Woods *et al.*, 2016 ▸; Kirsch *et al.*, 2019 ▸). Here, we screened our in-house fragment library, consisting of ∼1130 fragments, against *Burkholderia pseudomallei* disulfide bond-forming protein A (BpsDsbA) using nuclear magnetic resonance (NMR) spectroscopy and X-ray crystallography. This enabled us to obtain structural information on the binding site and the binding interactions between the fragment ligands and the protein.

The oxidoreductase disulfide bond-forming protein A (DsbA) is required for the correct folding of multiple virulence factors such as the type 3 secretion system, diverse proteases, flagellar proteins and many other virulence-associated proteins in bacteria (Heras *et al.*, 2009 ▸; Coulthurst *et al.*, 2008 ▸; Ireland *et al.*, 2014 ▸; Bocian-Ostrzycka *et al.*, 2017 ▸; Smith *et al.*, 2016 ▸). DsbA works in tandem with its membrane-embedded partner protein DsbB, which is required to maintain DsbA in its active, oxidized state. Deletion of the DsbA gene (*ΔdsbA*) is not lethal for bacteria such as *Escherichia coli* (Bardwell *et al.*, 1991 ▸), *Shigella flexneri* (Yu, 1998 ▸), *Francisella tularensis* (Qin *et al.*, 2011 ▸; Ren *et al.*, 2014 ▸) and *B. pseudo­mallei* (Ireland *et al.*, 2014 ▸; McMahon *et al.*, 2018 ▸), although mutants display phenotypes such as reduced motility, reduced adhesion and a decreased ability to replicate inside a host. Many of these phenotypes are due to the misfolding of a disulfide-containing protein in the absence of DsbA. These characteristics make DsbA an attractive target for antivirulence drug discovery, a strategy that aims to disarm rather than kill bacteria. Such a strategy may be beneficial in reducing the selective pressure for the development of resistance (Allen *et al.*, 2014 ▸; Heras *et al.*, 2015 ▸; Mühlen & Dersch, 2016 ▸; Smith *et al.*, 2016 ▸; Bocian-Ostrzycka *et al.*, 2017 ▸).

*B. pseudomallei* is a Gram-negative bacterium that is found predominantly in tropical areas and is the causative agent of the deadly disease melioidosis (Wiersinga *et al.*, 2018 ▸). Infections by this pathogen often result in severe illness or death, even after intensive antibiotic treatment (Dance, 2014 ▸; Schweizer, 2012 ▸; Rhodes & Schweizer, 2016 ▸). *B. pseudomallei* is intrinsically resistant to many currently available antibiotics, so that treatment of infection is prolonged and expensive, often requiring intravenous antibiotics for up to two weeks followed by oral antibiotics for several months (Currie, 2015 ▸).

Deletion of *dsbA* or *dsbB* results in the attenuation of *B. pseudomallei* virulence, and the deletion mutants have reduced protease activity and reduced motility. Importantly, mice infected with the deletion mutants have significantly increased survival rates in infection models compared with mice infected with wild-type *B. pseudomallei* (Ireland *et al.*, 2014 ▸; McMahon *et al.*, 2018 ▸).

BpsDsbA is an oxidoreductase enzyme that has been biochemically characterized and its structure determined to a resolution of 1.9 Å (Ireland *et al.*, 2014 ▸). The structure revealed a relatively featureless active-site surface with shallow pockets and a significantly shortened hydrophobic groove compared with *E. coli* DsbA (EcDsbA; Ireland *et al.*, 2014 ▸; McMahon *et al.*, 2014 ▸), suggesting that it may be challenging to find small-molecule inhibitors of BpsDsbA.

Techniques such as NMR, surface plasmon resonance (SPR; Adams *et al.*, 2015 ▸) and crystallography (Smith *et al.*, 2016 ▸; Duncan *et al.*, 2019 ▸) have all been used to identify small molecules that bind to EcDsbA, and some of these small molecules also inhibit EcDsbA in activity-based assays (Halili *et al.*, 2015 ▸; Totsika *et al.*, 2018 ▸; Mohanty *et al.*, 2017 ▸). Although inhibitors and small-molecule screening have mostly focused on EcDsbA, there has also been some success in identifying molecules binding to BpsDsbA (Nebl *et al.*, 2020 ▸; McMahon *et al.*, 2018 ▸). A short peptide derived from the sequence of its partner protein BpsDsbB has been shown to bind BpsDsbA using crystallography, revealing a relatively flat interaction site around the active site of the protein (McMahon *et al.*, 2018 ▸). Additionally, a fragment was shown to bind at a conformationally dynamic site on the surface of the protein using NMR (Nebl *et al.*, 2020 ▸).

In this work, we report two fragments that bind to BpsDsbA which could potentially be suitable for further development as inhibitors. These are bromophenoxy propanamide (**1**) and 4-methoxy-*N*-phenylbenzenesulfonamide (**2**). Binding was characterized using NMR and X-ray crystallo­graphy. Both **1** and **2** bind to a transient (or ‘cryptic’) pocket on BpsDsbA located adjacent to the redox active site which is not observed in the apo BpsDsbA structure. This cryptic pocket is formed by a shift in the side-chain conformation of a tyrosine residue to accommodate the fragments.

## 2. Materials and methods

### 2.1. Protein expression and purification for crystallization and peptide-oxidation assay

Recombinant BpsDsbA was expressed as described by Ireland *et al.* (2014 ▸). Briefly, plasmids with the BpsDsbA gene in a modified pET22 vector with a Tobacco etch virus protease (TEV) cleavage site followed by a His_6_ metal-affinity tag were transformed into *E. coli* BL21(DE3)pLysS competent cells, grown in 10 ml lysogeny broth (LB) containing chloramphenicol (CAM) and ampicillin (AMP), and incubated at 37°C overnight. Pre-cultures were used to start a 1 l culture in autoinduction medium also containing CAM and AMP (Studier, 2005 ▸). pET-28a plasmids containing the BpsDsbB gene with a noncleavable His_8_ tag were used to transform *E. coli* C41 cells specialized in membrane-protein expression, also using autoinduction medium supplemented with kanamycin.

BpsDsbA was purified according to the protocol described by Ireland *et al.* (2014 ▸). In brief, after expression the cells were harvested by centrifugation at 6000*g*. The pellet was resuspended in buffer consisting of 25 m*M* tris(hydroxymethyl)­aminomethane (Tris) pH 7.5, 150 m*M* NaCl. The cells were lysed by two passages at 165 MPa in a cell disrupter (Constant Systems) and the debris was separated from the supernatant containing the soluble protein by centrifugation (30 min at 30 000*g*). Imidazole (pH 7.5) was then added to the supernatant to a final concentration of 5 m*M* and the solution was subjected to immobilized metal-affinity chromatography (IMAC) by incubation with TALON cobalt resin (Takara) for 1 h at 4°C. The resin-bound protein was loaded onto a gravity-flow column and washed with 2 × 5 column volumes (CV) of wash buffer (10 m*M* imidazole, 500 m*M* NaCl, 25 m*M* Tris pH 7.5) before elution in 5 CV of 300 m*M* imidazole, 150 m*M* NaCl, 25 m*M* Tris pH 7.5. The protein was buffer-exchanged to remove imidazole using a 16/260 HiLoad desalting column (GE Healthcare). BpsDsbA was then incubated with TEV protease in a 1:50 (TEV:BpsDsbA) stoichiometric ratio overnight at 4°C. The next day, the cleaved His_6_ tags, noncleaved protein and TEV protease (also His_6_-tagged) were removed by reverse IMAC in TALON resin, with the target protein in the flowthrough. The protein was oxidized by mixing it with a molar excess of oxidized glutathione (GSSG) at room temperature for 1 h (50:1 stochiometric ratio of GSSG:BpsDsbA) and the oxidation state of the protein was monitored using the Ellman test (Ellman, 1959 ▸). A final size-exclusion step in 25 m*M* 4-(2-hydroxyethyl)-1-piperazine­ethanesulfonic acid (HEPES) pH 7.5, 150 m*M* NaCl was used to remove GSSG and impurities. The fractions corresponding to the protein were pooled, concentrated to 33 mg ml^−1^ using an Amicon Ultra 50 ml 10 kDa molecular-weight cutoff centrifugal filter (Merck Millipore) and then aliquoted before flash-freezing the protein sample in liquid nitrogen. Protein concentration was estimated using a NanoDrop ND-1000 spectrophotometer (Thermo Scientific).

Membrane preparations of BpsDsbB for the peptide-oxidation assay were generated using a method similar to that reported by Christensen *et al.* (2019 ▸). Briefly, the gene for BpsDsbB (UniProt ID Q63RY4) was inserted into a pET-28a plasmid in front of a sequence coding for a C-terminal non­cleavable His_8_ tag. The plasmid was inserted into *E. coli* C41 cells specialized for the expression of membrane proteins (Wagner *et al.*, 2008 ▸), which were grown in autoinduction medium (Studier, 2005 ▸) for 24 h at 30°C with shaking at 220 rev min^−1^. The cells were harvested by centrifugation at 6000*g* and were resuspended in phosphate-buffered saline (PBS). The cells were disrupted by two passages at 207 MPa through a cell disruptor (Constant Systems). Large debris was removed by centrifugation for 15 min at 15 000*g* and membranes containing protein were further separated from solution by ultracentrifugation for 1 h 15 min at 180 000*g.* The membrane pellet was resuspended in PBS prior to use in the peptide-oxidation assay.

### 2.2. Expression and purification of [U-^15^N]-BpsDsbA for NMR spectroscopy

Uniformly ^15^N-labelled ([U-^15^N]) BpsDsbA was expressed at the National Deuteration Facility (NDF), Australian Nuclear Science and Technology Organisation (ANSTO). The gene encoding BpsDsbA was inserted into a pET-24a vector maintaining the TEV protease-cleavable N-terminal His_6_ tag for protein expression using a high cell-density protocol as reported previously (Duff *et al.*, 2015 ▸). Briefly, 300 µl freshly transformed *E. coli* BL21 Star (DE3) cells were inoculated into 10 ml H_2_O ModC1 minimal medium and incubated overnight at 30°C with shaking at 220 rev min^−1^. This cell suspension was diluted fivefold in fresh ^1^H,^15^N-ModC1 medium (40 g l^−1^ glycerol, 5.16 g l^−1^ ^15^NH_4_Cl, ≥98 atom% ^15^N) and grown at 37°C for two OD_600_ doublings. Finally, the cells were inoculated into fresh ^1^H,^15^N-ModC1 to a volume of 100 ml and grown to an OD_600_ of 0.9 before inoculation into 900 ml labelled expression medium as described in a 1 l working volume bioreactor. The *E. coli* cells were grown at 25°C until the OD_600_ reached 14.8 and expression was induced by the addition of isopropyl β-d-1-thiogalactopyranoside (IPTG) to a final concentration of 1 m*M*. After 22.5 h induction at 20°C, during which a further 5.1 g ^15^NH_4_Cl was added to the culture, the labelled cell suspension was pelleted by centrifugation at 8000*g* for 20 min and the pellet was stored at −80°C.

BpsDsbA purification was performed in-house using the protocol reported previously by Nebl *et al.* (2020 ▸). Briefly, the frozen cell pellet was resuspended in lysis buffer comprising 50% BugBuster MasterMix (Novagen) and 50% buffer *A* consisting of 20 m*M* HEPES, 100 m*M* NaCl, 10 m*M* imidazole pH 8.0, using 2.5 ml per gram of cell pellet. One EDTA-free protease-inhibitor tablet (Roche) was added to the lysis buffer to prevent proteolysis. The mixture was agitated for 30 min at room temperature. To ensure complete cell lysis, sonication was performed on ice for 7 × 30 s at 50% duty cycle. The lysate was centrifuged at 75 465*g* for 30 min at 4°C. The supernatant was filtered through a 0.22 µm syringe filter and loaded onto an immobilized Ni^2+^-affinity column (HisTrap HP 5 ml, GE Healthcare) using buffer *A* and eluted using a gradient of 10–500 m*M* imidazole. Fractions containing the target protein were pooled and exchanged back to 100% buffer *A* using a Sephadex desalting column (HiPrep 26/10 column, GE Healthcare). TEV cleavage was performed overnight at 23°C with 1 m*M* DTT and 0.1 mg TEV per 10 mg of protein. A second reverse IMAC step was performed to collect the TEV-cleaved protein and remove His-tagged TEV protease, cleaved His_6_ tag and uncleaved BpsDsbA. The TEV-cleaved BpsDsbA was oxidized overnight at 4°C using freshly prepared copper phenanthroline at a final concentration of 1.5 m*M*. A final desalting step was performed to remove copper phenanthroline and exchange the sample into 50 m*M* HEPES, 50 m*M* NaCl, 2 m*M* EDTA pH 6.8 prior to purification by size-exclusion chromatography using a gel-filtration column (HiLoad 26/60 Superdex 75 column, GE Healthcare). The sample was concentrated using a 10 kDa molecular-weight cutoff centrifugal filter (Merck Millipore). The protein concentration was estimated using a NanoDrop 1000 spectrophotometer (Thermo Scientific). Finally, 1 m*M* phenylmethylsulfonyl fluoride (PMSF), 0.02% NaN_3_ and 10% D_2_O were added to the protein stock prior to NMR experiments.

### 2.3. Acquisition of small-molecule fragments

Bromophenoxy propenamide (**1**) (≥95% purity) was purchased from hit2lead (Chembridge Corporation, San Diego, California, USA).

4-Methoxy-*N*-phenylbenzenesulfonamide (**2**) was synthesized according to a literature procedure (Bernar *et al.*, 2018 ▸). Further details are given in the supporting information (Supplementary Fig. S1).

### 2.4. Quality control and solubility assessment of **1** and **2** in aqueous NMR buffer

The solubility of **1** and **2** was assessed by recording a set of 1D ^1^H-NMR spectra in aqueous NMR buffer (50 m*M* HEPES, 25 m*M* NaCl, 2 m*M* EDTA, 2% d_6_-DMSO, 100 µ*M* DSS, 10% D_2_O at pH 6.8). Chemical shifts and peak volumes of individual proton signals in the 1D ^1^H spectra were measured in order to identify possible aggregation either via concentration-dependent changes in the chemical shifts of the peaks or deviation from the expected concentration-dependent increase in peak volume (LaPlante *et al.*, 2013 ▸). 1D ^1^H spectra were collected on a 600 MHz spectrometer equipped with a CryoProbe at 298 K with a relaxation delay of 10 s. 1D ^1^H spectra were processed and analyzed using *MNova* (Bernstein *et al.*, 2013 ▸).

### 2.5. Chemical shift perturbation analysis and estimation of ligand-binding affinity (*K*_d_) by 2D [^15^N,^1^H]-HSQC NMR

The binding affinity of **1** and **2** for oxidized BpsDsbA was assessed by titration against 100 µ*M* ^15^N-labelled BpsDsbA. Backbone assignments of both redox states of BpsDsbA have been reported previously by Nebl *et al.* (2020 ▸); these assignments were used for the chemical shift perturbation (CSP) analysis in the 2D [^15^N,^1^H]-heteronuclear single-quantum coherence (HSQC) spectra using either *CARA* (Keller, 2005 ▸; http://cara.nmr.ch) or *SPARKY* (Lee *et al.*, 2015 ▸). Fragments **1** and **2** were titrated at concentrations of 0.25, 0.50, 1 and 2 m*M* with 100 µ*M* [U-^15^N]-BpsDsbA in NMR buffer (50 m*M* HEPES, 25 m*M* NaCl, 2 m*M* EDTA, 2% d_6_-DMSO, 1 m*M* PMSF, 10% D_2_O at pH 6.8). CSPs were calculated for each perturbed peak according to (1) (Nebl *et al.*, 2020 ▸), 

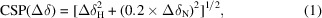

where Δδ_H_ and Δδ_N_ are the measured differences between the chemical shifts in the free versus bound spectra for the hydrogen and nitrogen signals (in p.p.m.), respectively. In an effort to estimate the dissociation constants (*K*_d_) of fragment **1** and fragment **2**, the CSP titration data were fitted to a one-site binding model in *GraphPad Prism* using nonlinear regression with (2) (Nebl *et al.*, 2020 ▸),

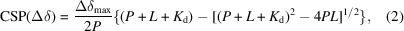

where *P* and *L* are the total concentrations of protein and ligand, respectively, Δδ_max_ is the maximum CSP upon saturation and *K*_d_ is the calculated dissociation constant. However, the CSP responses were observed to increase linearly with concentration, and so reliable estimates of *K*_d_ could not be obtained. These data do provide an indication of the site of interaction between the ligand and oxidized BpsDsbA by plotting the CSP magnitude as a gradient onto the crystal structure of BpsDsbA.

### 2.6. Crystallization of BpsDsbA for soaking experiments

Oxidized BpsDsbA, purified in 25 m*M* HEPES with 150 m*M* NaCl, was concentrated to 25–33 mg ml^−1^, dispensed in 100 nl drops onto a MRC-2 96-well sitting-drop plate (Hampton Research) and mixed with 100 nl crystallization buffer (0.1 *M* HEPES pH 7.5, 0.2 *M* Li_2_SO_4_ and a 28–34% gradient of PEG 3350). Crystal needles typically appeared after several hours and continued to grow for 4–5 days. The typical needle-crystal length was 70–300 µm, with a width of 20–50 µm. Fragments were dissolved in DMSO to a final concentration of between 5 and 25 m*M*. The fragment–DMSO solution was mixed with the crystallization buffer to final concentrations ranging from 0.25 to 1.25 m*M* and a BpsDsbA crystal was soaked in the fragment solution for approximatively 2 h. Similarly, crystals used to generate the background *Pan-Dataset Density Analysis* (*PanDDA*) map (Pearce, Krojer, Bradley *et al.*, 2017 ▸) were soaked in mother liquor containing 5% DMSO without fragments for 2 h. After soaking, crystals were fished out using nylon loops and cryocooled in liquid nitrogen (the high concentration of PEG in the mother liquor acted as a cryoprotectant).

### 2.7. Co-crystallization of BpsDsbA with **1** or with **2**

BpsDsbA was purified and oxidized as described above, concentrated to 33 mg ml^−1^, mixed with 10 m*M* **1** and kept on ice for 2 h. The solution was centrifuged to remove excess fragment that did not dissolve. A 100 nl drop of solution containing the protein in the presence of **1** was then dispensed in hanging drops and combined with a 100 nl drop of mother solution from commercial screens at 20°C using a Mosquito robot (SPT Labtech). Crystal needles grew in 60% Tacsimate (a mixture of malonate, citrate, succinate, malate, acetate, formate and tartrate from Hampton Research; McPherson & Cudney, 2006 ▸) after a few hours and continued to grow over 2–3 days. A needle crystal approximately 800 µm in length and 70 µm in width was fished out with a nylon loop and cryoprotected in mother liquor diluted with ethylene glycol (EG) to a final EG concentration of 20%(*v*/*v*). The crystal was then cryocooled in liquid nitrogen and tested in an X-ray diffraction experiment.

Similarly, oxidized BpsDsbA at 33 mg ml^−1^ was mixed with a large molar excess of **2** and incubated on ice for 2 h. Once again the solution was centrifuged to remove excess fragment that did not dissolve. A 100 nl drop of solution containing protein in the presence of **2** was mixed with 100 nl crystallization solution, dispensed as a hanging drop onto an MRC-2 crystallization plate and incubated at 20°C. Long crystal needles growing up to 1000 µm in length and 100 µm in width appeared after 2–3 days in 0.1 *M* HEPES pH 7.5, 0.2 *M* Li_2_SO_4_, 29.5% PEG 3350. Crystals were fished out with nylon loops and flash-cooled without additional cryoprotection.

### 2.8. X-ray diffraction experiments and refinement

X-ray diffraction data were collected at 100 K at the Australian Synchrotron, which is part of the Australian Nuclear Science and Technology Organisation (ANSTO), on the macromolecular crystallography beamlines MX1 (ADSC Quantum 210r Detector) and MX2 (EIGER 16M detector, funded by the Australian Cancer Research Foundation). Data were indexed, scaled and analyzed with the *autoPROC* pipeline (Vonrhein *et al.*, 2011 ▸) where possible, or manually with *XDS* (Kabsch, 2010 ▸) when *autoPROC* analysis failed. Structures were solved by molecular replacement using the oxidized BpsDsbA model with PDB code 4k2d (Ireland *et al.*, 2014 ▸) and refined using the *DIMPLE* pipeline, which is part of *CCP*4 (Winn *et al.*, 2011 ▸). Occasionally data sets required a stepwise analysis, in which case *Phaser* (McCoy *et al.*, 2007 ▸) and *phenix.refine* (Afonine *et al.*, 2012 ▸; Liebschner *et al.*, 2019 ▸) were used. Structures were then manually inspected with *Coot* (Emsley *et al.*, 2010 ▸) and *MolProbity* (Chen *et al.*, 2010 ▸). Refinement steps were repeated as required, alternating between *Coot* and *phenix.refine*. Ligand coordinates were generated from SMILES files using *eLBOW* (Moriarty *et al.*, 2009 ▸). Initial inspection of the data sets did not suggest density indicative of ligand binding. *PanDDA* (*pandda.analyse*) was run on the Griffith University high-performance cluster (HPC) ‘Gowonda’ following the instructions at https://pandda.bitbucket.io/pandda/tutorials.html. ‘Hits’ were inspected with *PanDDA* (*pandda.inspect*) through the *Coot* interface. The majority of these hits were false positives. Fragments **1** and **2** were identified as hits and further refined with *phenix.refine* and *Coot*. The models used in the *PanDDA* analysis, the corresponding MTZ files and the fragment CIF files, when present, were deposited in Zenodo (https://doi.org/10.5281/zenodo.5480892). The prefixes of the folders indicate whether they contain a ground-state data set (APO) used to generate the background map or a data set from a crystal soaked with fragment (LIG).

The structures of BpsDsbA co-crystallized with fragments **1** or **2** were solved by molecular replacement with *DIMPLE* (Winn *et al.*, 2011 ▸) and *Phaser* (McCoy *et al.*, 2007 ▸) using the oxidized BpsDsbA structure (PDB entry 4k2d) as a search model. Models were refined using *phenix.refine* (Afonine *et al.*, 2012 ▸; Liebschner *et al.*, 2019 ▸) and *Coot* (Emsley *et al.*, 2010 ▸), and *MolProbity* (Chen *et al.*, 2010 ▸) was used for validation of the protein structure. The placement of fragments **1** and **2** was validated with the script giant.score_model, which is part of *PanDDA* (Pearce, Krojer, Bradley *et al.*, 2017 ▸), available as part of *CCP*4 (Winn *et al.*, 2011 ▸).

### 2.9. Peptide-oxidation assay

The ability of fragments **1** and **2** to inhibit BpsDsbA was tested in a peptide-oxidation assay as described previously by Halili *et al.* (2015 ▸). Briefly, a synthetic peptide with two fluorescent groups at each extremity and two cysteines near each end can be oxidized in the presence of active DsbA. Upon oxidation, the two fluorescent groups are brought into close contact and can be excited at 340 nm to fluoresce at 615 nm. During the typical uninhibited reaction, the fluorescence of the peptide increases over 10–15 min until a plateau is reached. In the presence of BpsDsbA inhibitors, the enzyme fails to oxidize the peptide and the fluorescence does not increase over time.

Samples were prepared in 384-well plates with final reactant concentrations of 60 n*M* BpsDsbA, 1.6 µ*M* BpsDsbB in membranes, fragment in the range from 0 to 20 m*M* and 10 µ*M* substrate peptide in a final volume of 50 µl. The reaction was monitored using a Synergy H1 Hybrid plate reader (Biotek) with the excitation wavelength set to 340 nm, emission to 620 nm and a 100 µs delay between excitation and reading. Plates were monitored for 3 h until a reaction plateau was reached.

## 3. Results

### 3.1. Identification of fragments binding to oxidized BpsDsbA using crystal-soaking experiments and *PanDDA* analysis

An initial screen of ∼1130 fragments obtained from the Monash Institute for Pharmaceutical Science (MIPS) fragment libraries (Doak *et al.*, 2014 ▸) was performed using ligand-detected saturation-transfer difference (STD) NMR (Mayer & Meyer, 1999 ▸) against the oxidized BpsDsbA and EcDsbA proteins (Nebl *et al.*, 2020 ▸; Adams *et al.*, 2015 ▸). A set of fragments was initially identified as binding to BpsDsbA by STD-NMR. These hits were considered to be validated if they elicited detectable CSP in protein-detected 2D [^15^N,^1^H]-HSQC spectra of BpsDsbA (Nebl *et al.*, 2020 ▸). Among these promising candidates, a small subset of fragments was selected for further analysis in this study.

**Figure 1:**
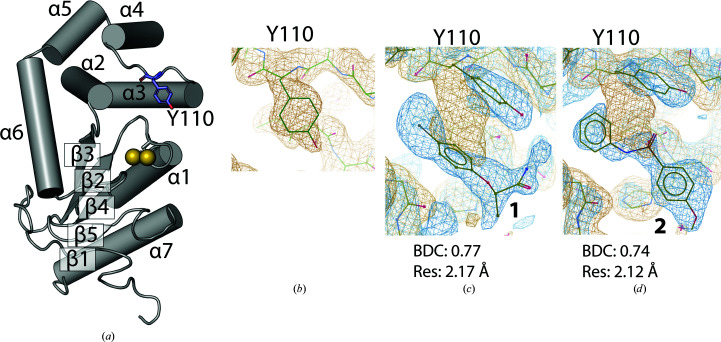
Event map generated by *PanDDA* around Tyr110 and fragments **1** and **2**. (*a*) Architecture of the apo BpsDsbA structure (PDB entry 4k2d; Ireland *et al.*, 2014 ▸) represented as a cartoon. α-Helices and β-strands are numbered α1–α7 and β1–β5, respectively. The active-site cysteines are indicated by yellow spheres; Tyr110 is represented in blue in stick format. (*b*) Close-up of the orientation of Tyr110 in the apo structure (no ligand present) and (*c*) and (*d*) in the presence of **1** and **2**, respectively. The Tyr110 side chain rotates to the right in this orientation (viewed along the C^β^—C^γ^ bond) towards helix α3 compared with the apo structure. This shift opens a small hydrophobic pocket into which the fragment binds. The reference apo 2*mF*_o_ − *DF*_c_ map, contoured at 1 r.m.s.d. and shown in orange, is the result of averaging 32 electron-density maps of apo BpsDsbA. The *PanDDA* event maps are shown in blue and are contoured at 2 r.m.s.d. for **1** and **2** in (*c*) and (*d*), respectively. These maps are estimates of the ligand-bound state (Pearce, Krojer, Bradley *et al.*, 2017 ▸). The background density correction (BDC) and the resolution (Res) are given for the event maps in (*c*) and (*d*).

A total of 29 unique fragments (Supplementary Fig. S2) were dissolved separately in 100% DMSO at concentrations of up to 25 m*M* and the solutions were used to soak individual BpsDsbA crystals. Crystals were exposed to X-rays either at the Australian Synchrotron (on the MX1 or MX2 beamlines) or on the laboratory source at The University of Queensland UQROCX crystallization facility. All of the crystals belonged to space group *P*2_1_2_1_2_1_, and all unit-cell angles were 90° as expected for this space group. All of the unit-cell dimensions were found to be between 59.0 and 60.0 Å for *a*, between 61.5 and 63.5 Å for *b* and between 68.0 and 70.5 Å for *c*. No interpretable positive difference Fourier density was picked up by *DIMPLE* in any of the data sets to indicate binding of the different fragments to the protein. We then reprocessed the diffraction data sets using a more sensitive method, *PanDDA* (Pearce, Krojer, Bradley *et al.*, 2017 ▸). We generated a background map from 32 X-ray diffraction data sets of the apoprotein soaked in DMSO (resolution ranging from 1.70 to 2.28 Å). This was used as the ‘ground-state’ model to reanalyze data sets of the protein soaked with the individual fragments. Using this method, we identified that two of the soaked-crystal data sets showed evidence for binding of fragment **1** with a background density correction (BDC) of 0.77 and fragment **2** with a BDC of 0.74, suggesting weak binding of the fragments (comparisons between raw maps and *PanDDA* maps are shown in in Supplenentary Fig. S3 and the chemical structures of fragments **1** and **2** are shown in Supplementary Figs. S4 and S5). In both models, the fragments bind near Tyr110, causing a change in the tyrosine side-chain position in comparison to the apo structure (Fig. 1 ▸ *b*). This shift revealed the presence of a small hydrophobic pocket into which each fragment binds (Figs. 1 ▸ *c* and 1 ▸ *d*). The binding of both fragments to BpsDsbA was then reproduced using independent co-crystallization experiments.

### 3.2. 2D [^15^N,^1^H]-HSQC NMR binding assay of **1** and **2** to BpsDsbA

Fragments **1** and **2** were previously identified as binding to oxidized BpsDsbA in an HSQC-NMR binding assay (Nebl *et al.*, 2020 ▸). The original HSQC screen was conducted using mixtures of two fragments. To confirm binding, we followed up the original experiment by recording 2D [^15^N,^1^H]-HSQC of BpsDsbA with each fragment individually.

**Figure 2:**
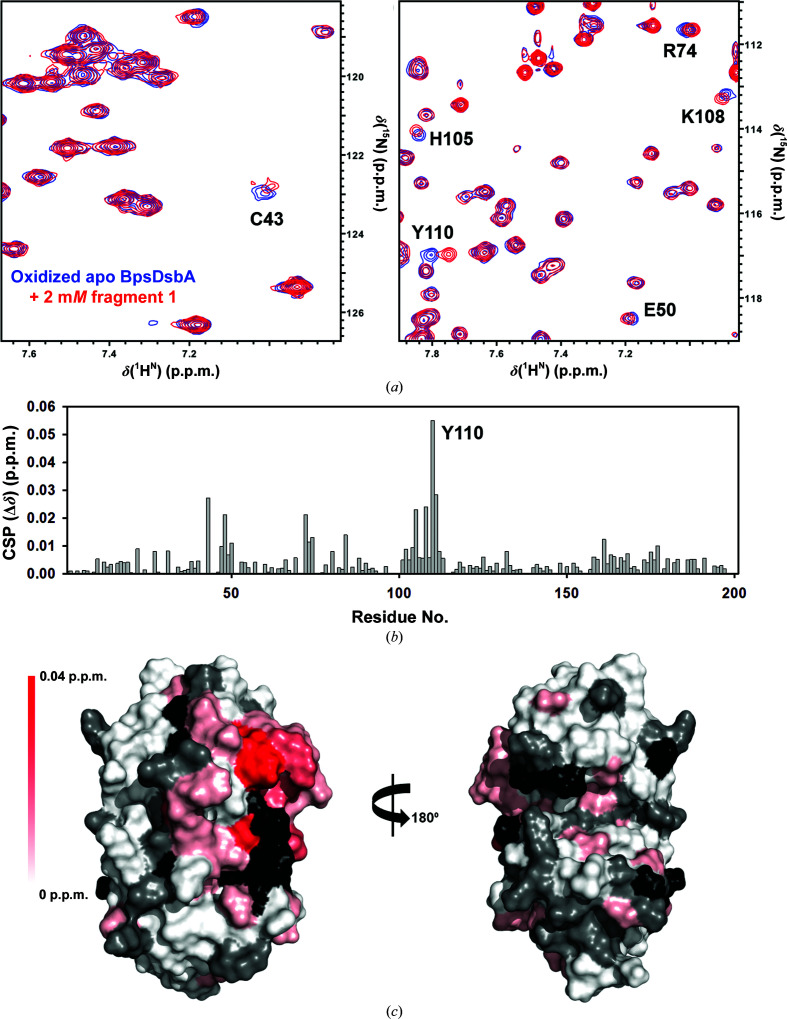
Characterization of bromophenoxy propanamide (**1**) binding to oxidized BpsDsbA by 2D [^15^N,^1^H]-HSQC NMR. (*a*) Expanded regions of the 2D [^15^N,^1^H]-HSQC data highlighting the backbone amide chemical shift perturbation (CSP) for selected residues of BpsDsbA without (blue) and with (red) 2 m*M* fragment **1**. (*b*) CSP observed for each BpsDsbA residue. (*c*) CSPs resulting from the addition of 2 m*M* fragment **1** are mapped onto the crystal structure of oxidized BpsDsbA (PDB entry 4k2d) as a colour gradient from red (CSP = 0.04 p.p.m.) to white (CSP = 0 p.p.m.). Non-shifting residues are shown in grey. Residues with unassigned amides and proline residues are shown in black. N-terminal residues (Ala1–Gly14) were removed for clarity.

**Figure 4:**
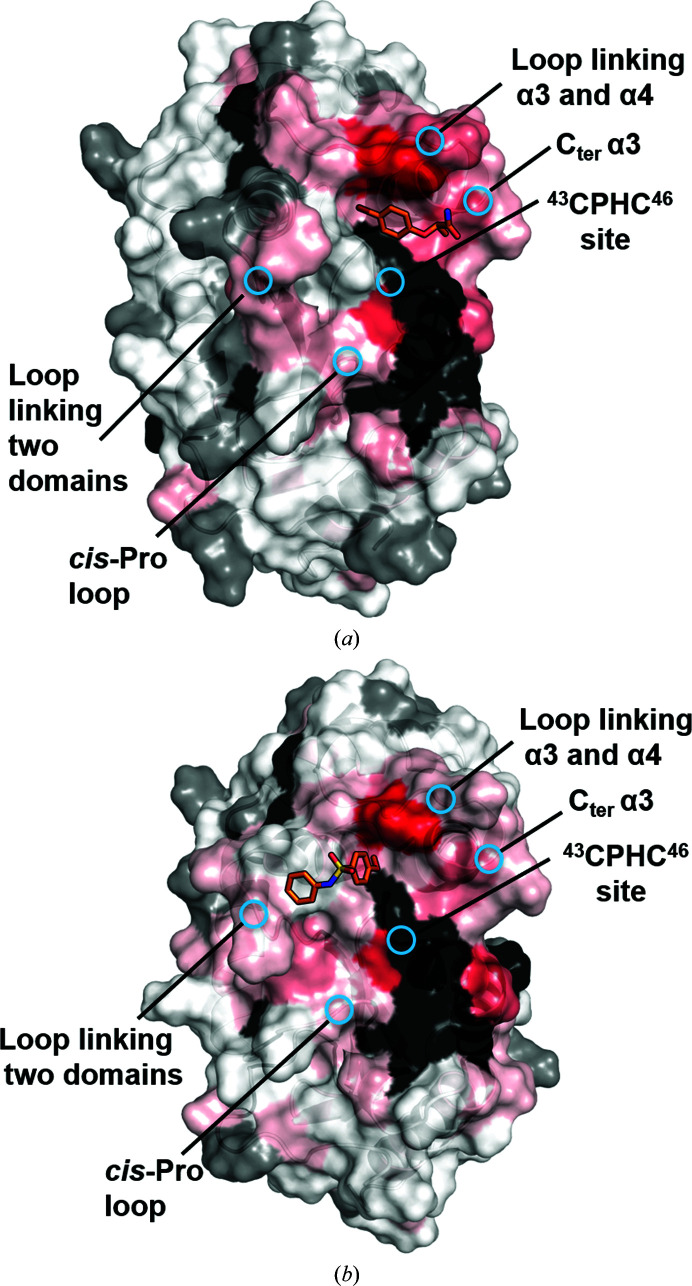
Characterization of fragments **1** and **2** binding to oxidized BpsDsbA by 2D [^15^N,^1^H]-HSQC NMR. Chemical shift perturbations (CSPs) resulting from the addition of 2 m*M* **1** (*a*) and 1 m*M* **2** (*b*) are mapped onto their corresponding complex crystal structures (PDB entry 7luh for **1** and chain *A* of PDB entry 7luj for **2**). CSPs are plotted as a colour gradient from red (CSP = 0.04 p.p.m.) to white (CSP = 0 p.p.m.). Residues with unassigned amides and proline residues are shown in black. Non-shifting residues are shown in grey. N-terminal residues (Ala1–Gly14) were removed for clarity. Blue circles highlight the specific region of interest on the protein.

Prior to HSQC screening of the two fragments, we evaluated their solubility in the NMR buffer (50 m*M* HEPES, 25 m*M* NaCl, 2 m*M* EDTA, 2% d_6_-DMSO, 100 µ*M* DSS, 10% D_2_O at pH 6.8). This confirmed that **1** and **2** were soluble in the NMR buffer (Supplementary Figs. S3 and S4). Overlays of the 2D [^15^N,^1^H]-HSQC spectra of oxidized BpsDsbA (100 µ*M*) in the absence and presence of **1** (2 m*M*) and **2** (1 m*M*) are shown in Figs. 2 ▸ and 3 ▸, respectively. CSPs resulting from the addition of **1** and **2** are mapped onto the crystal structure of oxidized BpsDsbA in Figs. 2 ▸ and 3 ▸ to provide a visual estimate of their binding sites. Both fragments produced backbone amide CSPs of >0.02 p.p.m. for residues Cys43, Glu48, His105, Tyr110 and Leu111. Two additional residues, Ala72 and Lys108, showed a CSP of >0.02 p.p.m. for **1**. These residues form a cluster between the ^43^CPHC^46^ active site, the *cis*-Pro loop adjacent to the active site, the C-terminal residues of helix α3, a loop connecting helix α3 and α4 and a loop between β3 and α2 connecting the two domains of the protein (Figs. 4 ▸ *a* and 4 ▸ *b*). The locations of the largest CSPs suggest that **1** and **2** may interact near the catalytic site of oxidized BpsDsbA; this site has previously been identified as a small-molecule binding site (Nebl *et al.*, 2020 ▸). The linear chemical shift trajectories upon increasing the fragment concentrations (Supplementary Fig. S6) indicate that the fragments are in fast exchange on the chemical shift time scale, suggesting weak binding (Ziarek *et al.*, 2011 ▸).

To estimate the binding affinity of fragments **1** and **2** to oxidized BpsDsbA, we recorded a series of [^15^N,^1^H]-HSQC spectra of 100 µ*M* BpsDsbA with increasing concentrations of fragments **1** (0–2 m*M*) and **2** (0–1 m*M*). For both fragments, the CSPs were observed to increase linearly with respect to concentration, and saturation was not achieved. Supplementary Fig. S7 shows the concentration-dependent CSP profiles of several binding-site residues. The CSP did not reach saturation at 2 m*M* ligand concentration, indicating that fragments **1** and **2** bind weakly with a *K*_d_ greater than the highest concentrations tested.

We previously observed redox-dependent ligand binding to BpsDsbA, and we hypothesized that this is due to differences in the dynamics of reduced and oxidized BpsDsbA (Nebl *et al.*, 2020 ▸). Here, we repeated the HSQC titrations of **1** and **2** against reduced BpsDsbA, and we did not observe any significant CSP (Supplementary Fig. S8). This indicates that fragments **1** and **2** bind preferentially to the oxidized form of BpsDsbA.

### 3.3. BpsDsbA co-crystallized with bromophenoxy propanamide (**1**) in a cryptic pocket binding site

Oxidized BpsDsbA was co-crystallized with **1** in 60% Tacsimate (Hampton Research); the resulting crystals diffracted to a resolution of 1.84 Å on beamline MX2 at the Australian Synchrotron and belonged to space group *P*2_1_2_1_2_1_. The structure was solved by molecular replacement using the original oxidized BpsDsbA structure (PDB entry 4k2d; Ireland *et al.*, 2014 ▸) as a search model. The structure was further refined by addition of the ligand, giving final *R*_work_ and *R*_free_ values of 16.5% and 19.4%, respectively (Table 1 ▸). Overall, the backbone structure (C^α^) of BpsDsbA in complex with **1** was very similar to that of the structure with no ligand, with a root-mean-square deviation (r.m.s.d.) of 0.14 Å between the residues of the two proteins (191 residues aligned with 191 residues using the *PyMOL* super function; Schrödinger).

**Figure 5:**
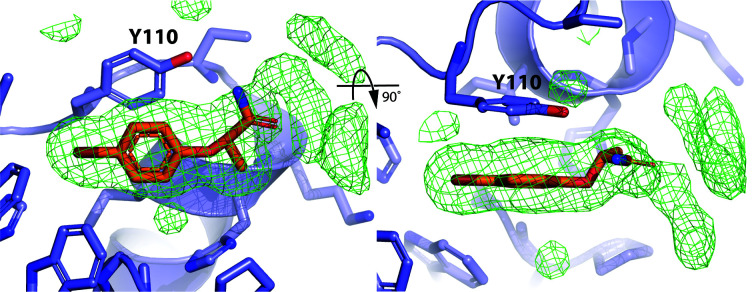
Polder map around bromophenoxy propanamide (fragment **1**). Left panel, side view of **1** and corresponding polder map at 1.84 Å showing a positive Fourier density peak at 3 r.m.s.d. surrounding the fragment. Right panel, 90° rotation of the same region. The density on the right of the fragment in this orientation could be due to the binding of water molecules or crystallant molecules or a combination thereof. The electron density for the Br atom is intense (present at a contour level of 20 r.m.s.d. in the polder map or beyond 8  r.m.s.d. in the typical 2*mF*_o_ − *DF*_c_ maps).

The data collected for BpsDsbA + fragment **1** (PDB entry 7luh) showed difference density corresponding to the ligand without the use of *PanDDA* and this was verified using a polder map (an OMIT map that accounts for solvent; Liebschner *et al.*, 2017 ▸). The polder map showed positive difference density for the ligand at a 3 r.m.s.d. contour level (Fig. 5 ▸). The signal was particularly intense for the Br atom, where difference density was visible even above the 20 r.m.s.d. level. Additional difference density was present near the modelled carboxamide of the fragment. This could be due to water, ethylene glycol or any of the smaller organic molecules found in the crystallization conditions (malonate, citrate, succinate, malic acid, acetate, formate and tartrate). We did not model any ligands into this density.

**Figure 6:**
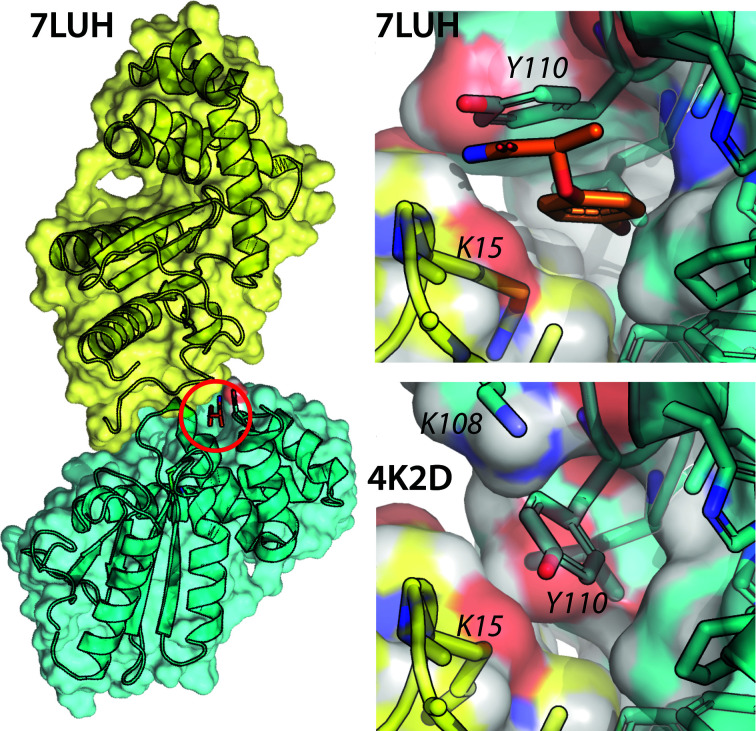
Crystal structure of BpsDsbA with bound bromophenoxy propanamide (fragment **1**). On the left, the protein organization in the crystal structure with PDB code 7luh is represented, with the ligand-binding pocket located at a crystal contact (the two protein monomers are coloured cyan and yellow). The ligand is shown in orange and the binding site is highlighted with a red circle. On the right, a close-up view of the ligand-binding pocket in PDB entry 7luh compared with the oxidized apo structure PDB entry 4k2d (Ireland *et al.*, 2014 ▸) is depicted. In the absence of fragment **1**, the hydrophobic pocket is occupied by the side chain of Tyr110. The residues lining the pocket on the symmetry-related copy of the protein are Val12, Ala13 and Lys15 (yellow). Proteins are shown as a combination of surface, cartoon and stick representations.

**Figure 7:**
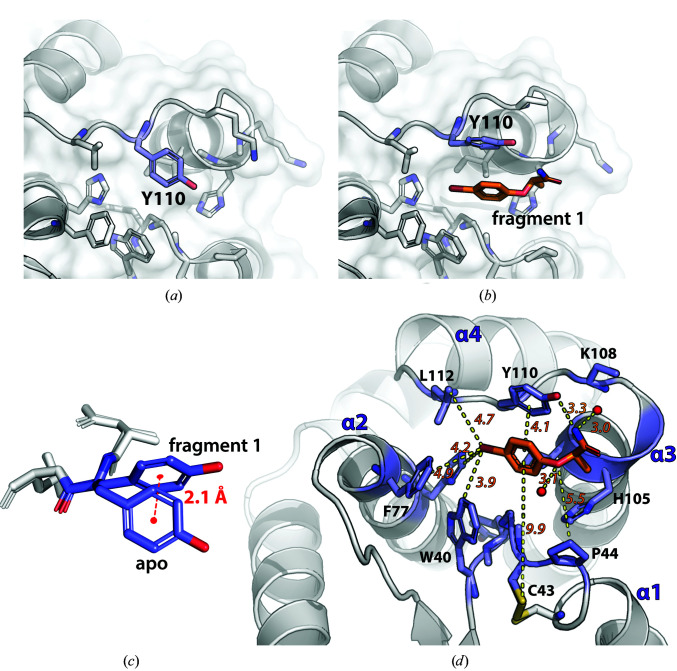
Bromophenoxy propanamide (**1**) binding requires a shift of Tyr110 to open a cryptic hydrophobic pocket. (*a*) Side-chain position of Tyr110 in the absence of ligand. (*b*) The same region in the presence of **1**, showing that Tyr110 shifts up (towards helix α3), opening a binding site for **1**. (*c*) Superposition of Tyr110 in apo and liganded conformations (fragment **1**): the centre of the benzene ring of the tyrosine is displaced 2.1 Å between the two conformations. (*d*) Structure of the BpsDsbA–fragment **1** complex (PDB entry 7luh) showing the cryptic pocket above the catalytic active site. Fragment **1** (orange) binds in a hydrophobic pocket. All residues within 5 Å of **1** are shown as purple sticks and labelled; water molecules are shown as red spheres. The distances between different atoms or ring centromeres separated by a yellow dashed line are given in italics (in Å). The S atoms in the ^43^CPHC^46^ active site of BpsDsbA are shown as yellow sticks. α-Helices are numbered. Note that Lys108 is truncated in PDB entry 7luh as no 2*mF*_o_ − *DF*_c_ density was visible beyond C^γ^ at 0.8 r.m.s.d.

Binding of fragment **1** accompanied a shift of more than 2 Å in the Tyr110 side chain from its orientation in the apo structure (measured from the centres of the aromatic rings of the two Tyr110 conformations), revealing a small hydrophobic pocket at the interface between two copies of the protein at the crystal contact (Fig. 6 ▸). The interactions between **1** and the protein are mostly hydrophobic, involving the side chains of Tyr110, Trp40, Phe77 and Leu112 from the original copy of the protein and Val12, Ala13 and Lys15 of the next symmetry-related protein molecule in the crystal. Additionally, there are π-stacking interactions between the aromatic rings of the fragment and Tyr110 (4.1 Å, measured from the centroid of each ring). The fragment binds within 10 Å of the redox active-site residue Cys43 (Fig. 7 ▸). During refinement of the structure, the optimal occupancy of fragment **1** was found to be 0.62, suggesting that the observed density reflects a mixture of the apo and fragment-bound forms of the protein in the crystal. Electron density near another region of the protein (located between Ser9 and Glu83) suggested the possibility of a second bound fragment **1**. However, the density was weak (visible at a low contour level of 0.6 r.m.s.d.) and there was no strong density indication for a Br atom. We also attempted to model a combination of malate/acetate/water, but could not identify a better model. On the basis of ligand validation statistics we chose not to model anything in this second electron density.

To assess the quality of the modelled fragment binding, ligand validation statistics were calculated as described by Pearce, Krojer & von Delft (2017 ▸). Briefly, several metrics are used together to determine the quality of the ligand placement: a real-space correlation coefficient (RSCC), with values varying from 0 (bad) to 1 (perfect), to determine how well the ligand fits the real-space electron density, a real-space *Z*-difference score (RSZD) that measures the accuracy of the model through local *Z*-difference distribution (Tickle, 2012 ▸), with values that can range from 0 (good) to ∞ (bad), and a real-space *Z*-observed score (RSZO) that measures the precision of the density by comparing the average electron density of residues with the map noise, with values ranging from 0 (bad) to ∞ (good) (Tickle, 2012 ▸). In addition, the *B*-factor stability of the ligand was calculated by comparing the ligand *B* factor with the *B* factor of neighbouring residues (see Pearce, Krojer & von Delft, 2017 ▸). For fragment **1** in PDB entry 7luh, the RSCC score is 0.97 (very good), the RSZD score is 0.9 (good), the model precision for fragment **1** (RSZO/occupancy) is 2.4 (good) and the *B*-factor stability is 1.35 (meaning that the ligand *B* factor is 1.35 times higher than the *B* factor of the nearby residues, which is high; see Supplementary Table S1 for all of the details).

### 3.4. BpsDsbA complexed with phenylbenzenesulfonamide (**2**) crystallizes with four molecules in the asymmetric unit

**Figure 8:**
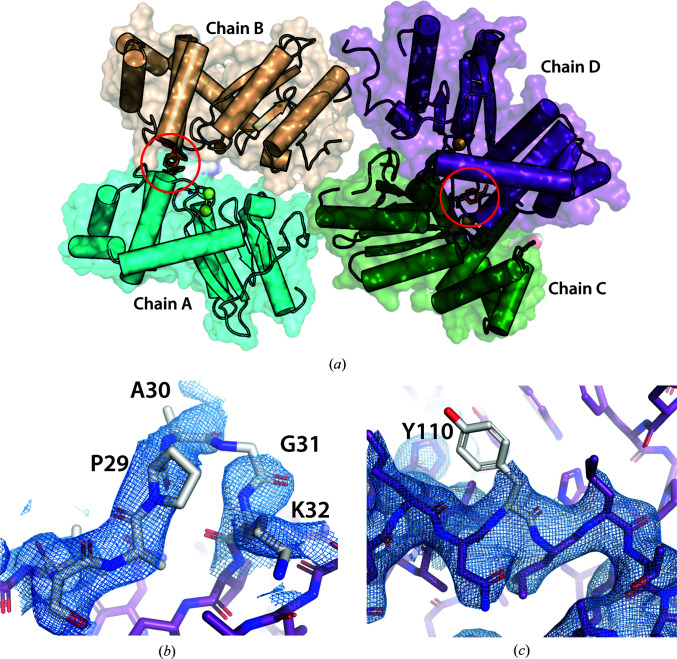
Structure of BpsDsbA crystallized with **2** (PDB entry 7luj). (*a*) Overall representation of the four molecules of BpsDsbA in the asymmetric unit. There are three copies of fragment **2**. Two copies bind near Tyr110 of chains *A* (cyan) and *B* (beige); they are partially overlapping and almost perfectly rotamerically symmetric around a *C*2 axis. The third copy of the ligand binds near Tyr110 of chain *D* (purple). No fragment was found in chain *C* (forest green). The position of the ligands are highlighted by red circles. The active-site S atoms are represented as spheres, and the protein chains are shown as cartoons with α-helices represented as cylinders for simplicity. (*b*, *c*) 2*mF*_o_ − *DF*_c_ electron densities at 2.31 Å resolution (contoured at 0.8 r.m.s.d., blue) around chain *D*. (*b*) The map around the loop between residue Pro29 and Lys32 (highlighted in grey) was not particularly sharp; single residues were difficult to fit in the densities and the electron density is discontinuous between Ala30 and Gly31. (*c*) Similarly, electron density was absent for the side chain of Tyr110 (note that fragment **2** was removed from this image for clarity).

Oxidized BpsDsbA was co-crystallized with **2** in a crystallization solution that typically generated crystals of the apoprotein, that is 200 m*M* Li_2_SO_4_, 100 m*M* HEPES pH 7.5, 29.5% PEG 3350. A crystal was harvested and diffracted to a resolution of 2.3 Å with space group *P*2_1_ and unit-cell parameters *a* = 69.4, *b* = 59.4 , *c* = 105.4 Å, α = γ = 90, β = 104.9° (Table 1 ▸). The structure was solved by molecular replacement using the oxidized apo BpsDsbA structure (PDB entry 4k2d; space group *P*2_1_2_1_2_1_) as a search model. One solution was found that included four copies of BpsDsbA per asymmetric unit (Fig. 8 ▸ *a*) and was refined to *R*_work_ and *R*_free_ values of 22.8% and 26.0%, respectively (Table 1 ▸). All four chains of the model align with each other with r.m.s.d.s between the residues of the different chains of below 0.3 Å (alignment of 188 residues with 188 residues for each pairwise comparison; r.m.s.d. chain *A*–chain *B* = 0.12 Å, r.m.s.d. chain *A*–chain *C* = 0.20 Å and r.m.s.d. chain *A*–chain *D* = 0.20 Å, measured using the *PyMOL* super function; Schrödinger). The backbone of chain *A* of this structure also aligns with the apo model PDB entry 4k2d with an r.m.s.d. of 0.24 Å (188 versus 191 residues aligned). The major difference between chain *A* and the apo model is the truncation of the three N-terminal residues of chain *A* in the data set of the complex relative to the published apo structure, which could not be modelled due to a lack of electron density to justify their placement. In chain *D*, electron density was poorly resolved for the side chains of residues in the loop 29–32 (Fig. 8 ▸ *b*) and residue Tyr110 that is reorientated in the presence of **2** (Fig. 8 ▸ *c*). Modelling of this residue is therefore tentative and must be interpreted with caution.

**Figure 9:**
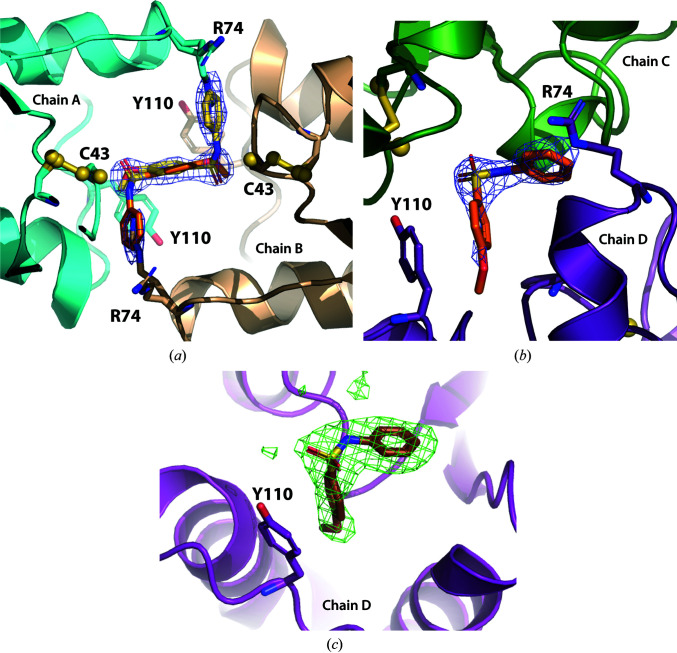
Electron-density maps for fragment **2**. (*a*) Fragment **2** modelled as two alternate binding modes (yellow and orange) at the interface of chains *A* (cyan) and *B* (beige) showing the 2*mF*_o_ − *DF*_c_ map at 0.8 r.m.s.d. in blue. The two modelled copies of the fragment are almost perfectly rotationally symmetric, and were modelled as alternate conformations of the same molecule. Similarly, Arg74 of chains *A* and *B* was built with alternate conformations. The two alternate copies of the fragment were refined to partial occupancies of 0.43 for conformation *A* (interacting with chain *A* of the protein) and 0.31 for conformation *B* (interacting with chain *B*). (*b*) 2*mF*_o_ − *DF*_c_ maps at 0.8 r.m.s.d. for fragment **2** modelled at the interface of chains *C* (forest green) and *D* (purple); the methoxy group of **2** is not well resolved but its position was confirmed by reference to the polder map. (*c*) Polder map (green) at a contour of 3.5 r.m.s.d. indicating the presence of the fragment. The bound model of fragment **2** binding to chain *D* was refined to a final occupancy of 0.88. All maps were calculated to a resolution of 2.3 Å. Residues are shown in stick format and labelled; other protein chains are shown in cartoon representation.

Although there are four copies of BpsDsbA in the asymmetric unit, the electron-density maps indicate that there are three molecules of fragment **2** bound between the four copies of the protein (Fig. 8 ▸ *a*). Two copies of fragment **2** are found between chains *A* and *B*; they are almost perfectly rotamerically symmetric and overlap in their methoxy­phenyl moieties (Fig. 9 ▸ *a*). As such they were modelled as alternative conformations of the same molecule (with occupancies of 0.43 and 0.31). In concert, the Arg74 side chains of protein chains *A* and *B* were modelled with two alternate conformations. When the fragment is present in the orientation where its methoxy­phenyl group binds near the Tyr110 side chain of protein chain *A* (orientation *A*), Arg74 of protein chain *A* (Arg74-*A*) is modelled with a conformation that avoids clashes with the fragment. In the absence of fragment **2** in orientation *A*, the Arg74-*A* side chain is modelled in a different conformation to occupy the position vacated by the fragment (Figs. 9 ▸ *a*, 9 ▸ *b* and 10 ▸ *d*). Similarly, we modelled Arg74 of chain *B* (Arg74-*B*) with two conformations which differ depending on the presence or absence of fragment **2** in orientation *B* (binding near the Tyr110 side chain of chain *B*). The two orientations *A* and *B* of fragment **2** sit in an almost completely closed pocket at the interface of protein chains *A* and *B* (Figs. 8 ▸ *a* and 10 ▸ *a*).

**Figure 10:**
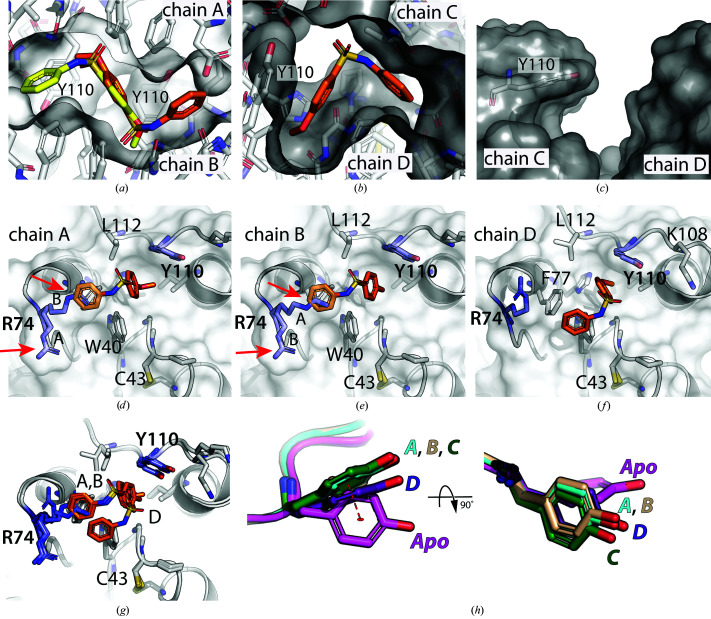
Interaction of phenylbenzenesulfonamide (**2**) with chains *A*, *B* and *D* of PDB entry 7luj. (*a*) Sectional view through the pocket at the interface of chains *A* and chain *B* (labelled) which tightly surrounds fragment **2** in orientations *A* (orange) and *B* (yellow). Tyr110 of both chains is visible behind the fragment, and the nonclashing alternate side-chain conformations of Arg74-*A* and Arg74-*B* were chosen to generate this figure. (*b*) Sectional view through the pocket at the interface of chains *C* and *D* (labelled): only one orientation of fragment **2** is observed in this pocket (orientation *D* in orange). Tyr110 of chain *D* is visible on the left of the fragment. (*c*) There is no fragment **2** binding near Tyr110 (grey stick) of chain *C* (labelled). This area is exposed to the solvent compared with Tyr110 of chains *A*, *B* and *D*. (*d*), (*e*) and (*f*) show each orientation of the fragment (*A*, *B* and *D*, respectively; fragment **2** is shown in orange) relative to their respective protein chain (labelled at the top left of each panel). The two alternate conformations of Arg74 are highlighted with red arrows in (*d*) and (*e*). In (*g*) all of the orientations are superposed together. Here it is apparent that orientations *A* and *B* of fragment **2** are very similar, while fragment **2** in orientation *D* is found slightly closer to the active-site residues. (*h*) compares the positions of the Tyr110 side chains of the different protein chains of PDB entry 7luj with Tyr110 of the apo structure, showing a 2 Å shift (shown as a red dotted line) between the middle of the tyrosine ring of chain *A* (cyan) and that of the apo structure.

The third copy of fragment **2** is located at the interface of chains *C* and *D*. Again the methoxy portion of the fragment binds near the side chain of Tyr110 of chain *D* (named orientation *D*), while the phenyl portion is located closer to the C*XX*C active site of protein chain *C* (Figs. 9 ▸ and 10 ▸ *b*). The occupancy of fragment **2** in orientation *D* was refined to a final value of 0.88. Again this copy of fragment **2** binds in an almost completely closed pocket (Fig. 10 ▸ *b*).

The 2*mF*_o_ − *DF*_c_ maps show binding of **2** in all three orientations (*A*, *B* and *D*) at an 0.8 r.m.s.d. contour level (Figs. 9 ▸ *a* and 9 ▸ *b*), although the density is better defined for the binding site involving chains *A* and *B*. The presence of the ligand near chain *D* was confirmed by polder map analysis (Fig. 9 ▸ *c*). Ligand validation statistics were calculated for fragment **2** (note that because fragment **2** was modelled as an alternative conformation of the same molecule between chains *A* and *B*, there are only two copies of the fragment to validate: orientation *A* and *B* together and orientation *D*). For the copy of the fragment binding between chains *A* and *B* the scores are RSCC = 0.9 (very good), RSZD = 0.2 (very good), RSZO/occupancy = 0.8 (low) and *B*-factor stability = 1.16 (OK). For the copy of fragment **2** in orientation *D* the values are RSCC = 0.84 (good), RSZD = 3.6 (high), RSZO/occupancy = 0.9 (low), *B*-factor stability = 1.07 (OK). Further details are provided in Supplementary Table S1.

Fragment **2** binds to chains *A*, *B* and *D* in a similar manner (Figs. 10 ▸ *d*–10 ▸ *g*) near Tyr110. However, there was no electron density indicating binding of fragment **2** near Tyr110 of chain *C*. In this chain, due to the arrangement of the protein chains in the crystal structure, Tyr110 is exposed to the solvent (Fig. 10 ▸ *c*). These findings suggest that binding of fragment **2** to a pocket near Tyr110 in the protein is relatively weak.

We also note that the side chain of Tyr110 adopts the same orientation in all four molecules in the asymmetric unit whether the fragment is bound (chains *A*, *B* and *D*) or not (chain *C*) (Fig. 10 ▸ *h*).

### 3.5. Fragments **1** and **2** do not inhibit the BpsDsbA–BpsDsbB redox cycle

Although the fragments bind weakly to BpsDsbA, we tested whether **1** or **2** were capable of inhibiting the enzymatic activity of BpsDsbA. This was evaluated in a peptide-oxidation assay using oxidized BpsDsbA. The assay uses a fluorescently labelled synthetic peptide with cysteines at either end. Oxidation of the substrate by BpsDsbA causes an increase in the fluorescence signal (Halili *et al.*, 2015 ▸). The reaction was monitored by measuring the increase in fluorescence over the first 10 min of the reaction, which is defined as the initial velocity. Inhibition is indicated by a decrease in the initial velocity compared with the control with no ligand present (addition of a matched concentration of DMSO only; Supplementary Fig. S9). Neither of the fragments exhibited any inhibitory activity in this assay; even at a maximum concentration of 20 m*M* the initial velocity of the reaction was comparable to that of the control reaction. This suggests that the weak binding affinity of the two fragments is not sufficient to compete with or inhibit the peptide used in this assay for binding to BpsDsbA.

## 4. Discussion

DsbA enzymes contribute to the virulence of many Gram-negative bacteria (Coulthurst *et al.*, 2008 ▸; Heras *et al.*, 2009 ▸; Ireland *et al.*, 2014 ▸; McMahon *et al.*, 2014 ▸), including the often-neglected pathogen *B. pseudomallei*. DsbA proteins have thus been identified as targets for therapeutic drugs (Bocian-Ostrzycka *et al.*, 2017 ▸; Allen *et al.*, 2014 ▸; Heras *et al.*, 2015 ▸; Smith *et al.*, 2016 ▸).

Several molecules have been reported that inhibit the activity of DsbA enzymes from *E. coli* (Adams *et al.*, 2015 ▸; Duprez *et al.*, 2015 ▸; Halili *et al.*, 2015 ▸), *Pseudomonas aeruginosa* (Mohanty *et al.*, 2017 ▸) and *Salmonella enterica* serovar Typhimurium (Totsika *et al.*, 2018 ▸). To date, only one small molecule has been reported to bind to oxidized BpsDsbA and inhibit the enzymatic activity *in vitro* (Nebl *et al.*, 2020 ▸). BpsDsbA has a shallow hydrophobic groove in comparison to EcDsbA, and a generally flatter surface (McMahon *et al.*, 2014 ▸), making it a more challenging drug target.

Here, we have reported the structure and binding interactions of two fragment molecules with oxidized BpsDsbA, both of which interact with a small, cryptic pocket close to the redox-active site of the protein. Both fragments, bromophenoxy propan­amide (**1**) and 4-methoxy-*N*-phenylbenzenesulfonamide (**2**), bound under Tyr110, which was shifted towards helix α3 compared with the apo structure of the protein (Figs. 7 ▸ and 10 ▸). Results were generated using both NMR and X-ray crystallo­graphy and support the findings of Nebl *et al.* (2020 ▸), who previously identified the presence of a cryptic pocket in the vicinity of Trp40, Cys43, Cys46, Arg74, Ile104, Tyr110 and Leu112.

The binding of fragments **1** and **2** to BpsDsbA is weak, with an NMR-estimated *K*_d_ of >2 m*M*. The weak binding is evident from the partial occupancy, high *B* factors and high *B*-factor stability of the modelled fragments in the crystal structures (Table 1 ▸). Fragment binding in the crystal structure may be affected by the low concentration of the fragment used in crystal soaking and co-crystallization experiments (1.25 m*M*) due to fragment solubility. For comparison, similar fragment-binding experiments using crystallography (Pearce, Krojer, Bradley *et al.*, 2017 ▸) typically use concentrations of ∼100 m*M*. Although the binding of the fragments is weak, the crystallo­graphic experiments identified that fragments **1** and **2** both bind in the same region of the protein, near Tyr110. A fragment-binding site near Tyr110 is supported by NMR experiments: a large CSP for Tyr110 is observed upon addition of fragments **1** and **2** to the protein.

The partial occupancies reported for the fragments in this paper may be overestimates that reflect superposition of the ligand and a conformation of Tyr110. The ligand validation statistics indicate that the fragments fit well in the real-space density (with an RSCC of >0.8 in every case); however, varying degrees of accuracy (RSZD) and *B*-factor stability suggest that the fragment alone might not describe the density perfectly.

We hypothesize that the weakly bound fragments were able to be observed in the crystal structure, despite the weak *K*_d_ and the low fragment concentration, because the binding site is enhanced by the arrangement of protomers in the crystal. Specifically, each of the fragment-binding sites is at a protein–protein interface in the crystal. Of note, in chain *C* of PDB entry 7luj, in which Tyr110 is exposed to solvent and more ‘open’, no electron density was observed for fragment **2**. The crystal arrangement may therefore have helped us to capture a very weak binding interaction using a low concentration of fragment.

The identification and characterization of the binding of fragments **1** and **2** to BpsDsbA is a key first step towards understanding this cryptic pocket and the dynamic behaviour of the active site at atomic resolution. This pocket is of interest because of its proximity to the active site, which suggests that expanding these fragments may generate more potent compounds that block the active site and inhibit the activity of BpsDsbA. The results presented here provide a starting point for the elaboration and further optimization of more potent small-molecule inhibitors for BpsDsbA using rational drug design. 

## Supplementary Material

PDB reference: BpsDsbA + fragment **1**, 7luh

PDB reference: BpsDsbA + fragment **2**, 7luj

Synthesis of 4-methoxy-N-phenylbenzenesulfonamide and Supplementary Figures. DOI: 10.1107/S2059798321011475/gm5084sup1.pdf

Supplementary Table S1. DOI: 10.1107/S2059798321011475/gm5084sup2.xlsx

Models used in PanDDA analysis, the corresponding MTZ files and fragment CIF files.: https://doi.org/10.5281/zenodo.5480892

## Figures and Tables

**Figure 3:**
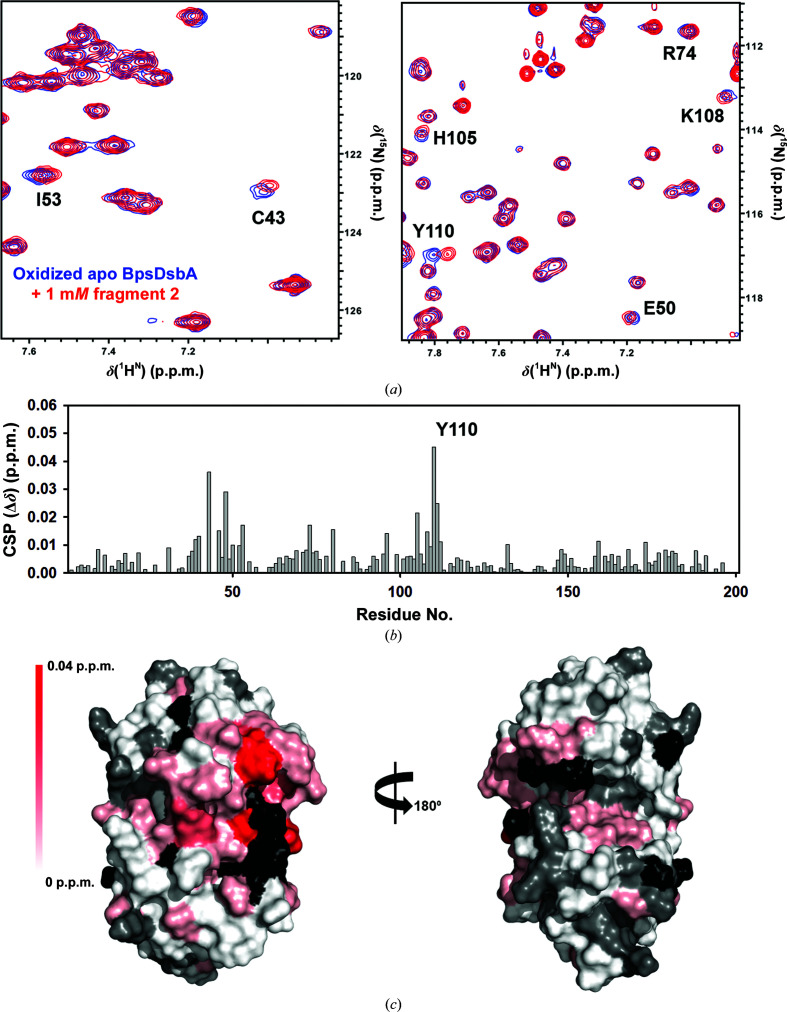
Characterization of 4-methoxy-*N*-phenylbenzenesulfonamide (**2**) binding to oxidized BpsDsbA by 2D [^15^N,^1^H]-HSQC NMR. (*a*) Expanded regions of the 2D [^15^N,^1^H]-HSQC data highlighting the backbone amide chemical shift perturbations (CSP) for selected residues of BpsDsbA without (blue) and with (red) 1 m*M* fragment **2**. (*b*) CSP observed for each BpsDsbA residue. (*c*) CSPs resulting from the addition of 1 m*M* **2** are mapped onto the crystal structure of oxidized BpsDsbA (PDB entry 4k2d) as a colour gradient from red (CSP = 0.04 p.p.m.) to white (CSP = 0 p.p.m.). Residues with unassigned amides and proline residues are shown in black. Non-shifting residues are shown in grey. N-terminal residues (Ala1–Gly14) were removed for clarity.

**Table 1:** Data-collection and refinement statistics Values in parentheses are for the highest resolution shell.

	BpsDsbA + **1** (PDB entry 7luh)	BpsDsbA + **2** (PDB entry 7luj)
No. of molecules in the asymmetric unit	1	4
Wavelength (Å)	0.9537	0.9537
Resolution range (Å)	36.7–1.84 (1.91–1.84)	38.4–2.31 (2.40–2.31)
Space group	*P*2_1_2_1_2_1_	*P*2_1_
*a*, *b*, *c* (Å)	59.5, 62.9, 69.4	69.4, 59.4, 105.4
α, β, γ (°)	90, 90, 90	90, 104.9, 90
Total reflections	154193 (15785)	241760 (21753)
Unique reflections	23090 (2249)	36226 (3064)
Multiplicity	6.7 (7.0)	6.7 (6.1)
Completeness (%)	99.23 (98.38)	97.40 (84.08)
Mean *I*/σ(*I*)	16.92 (1.51)	6.88 (1.05)
Wilson *B* factor (Å^2^)	31	45
*R*_merge_	0.071 (1.27)	0.172 (1.36)
*R*_meas_	0.077 (1.37)	0.187 (1.49)
*R*_p.i.m._	0.030 (0.512)	0.072 (0.592)
CC_1/2_	0.999 (0.633)	0.994 (0.573)
CC*†	1.000 (0.880)	0.999 (0.854)
Reflections used in refinement	23063 (2248)	35652 (3064)
Reflections used for *R*_free_	1986 (183)	1785 (137)
*R*_work_	0.165 (0.313)	0.228 (0.326)
*R*_free_	0.194 (0.344)	0.260 (0.342)
CC_work_	0.972 (0.836)	0.944 (0.606)
CC_free_	0.951 (0.768)	0.946 (0.567)
No. of non-H atoms		
Total	1747	6232
Macromolecules	1528	5940
Ligands	13	66
Solvent	206	233
Protein residues	191	756
R.m.s.d., bonds (Å)	0.008	0.004
R.m.s.d., angles (°)	0.84	0.6
Ramachandran favoured (%)	98.94	99.2
Ramachandran allowed (%)	1.06	0.8
Ramachandran outliers (%)	0.00	0.00
Rotamer outliers (%)	0.61	1.29
Clashscore	2.29	3.6
Average *B* factor (Å^2^)		
Overall	37	61
Macromolecules	36	61
Ligands	48	69
Solvent	42	54
No. of TLS groups	3	12

† CC* = [2CC_1/2_/(1 + CC_1/2_)]^1/2^ (Karplus & Diederichs, 2012 ▸).
